# Defining and Systematic Analyses of Aggregation Indices to Evaluate Degree of Calcium Oxalate Crystal Aggregation

**DOI:** 10.3389/fchem.2017.00113

**Published:** 2017-12-07

**Authors:** Sakdithep Chaiyarit, Visith Thongboonkerd

**Affiliations:** Medical Proteomics Unit, Office for Research and Development, Faculty of Medicine Siriraj Hospital; and Center for Research in Complex Systems Science, Mahidol University, Bangkok, Thailand

**Keywords:** aggregation assay, aggregation index, CaOx, COM, kidney stone, nephrolithiasis

## Abstract

Crystal aggregation is one of the most crucial steps in kidney stone pathogenesis. However, previous studies of crystal aggregation were rarely done and quantitative analysis of aggregation degree was handicapped by a lack of the standard measurement. We thus performed an *in vitro* assay to generate aggregation of calcium oxalate monohydrate (COM) crystals with various concentrations (25–800 μg/ml) in saturated aggregation buffer. The crystal aggregates were analyzed by microscopic examination, UV-visible spectrophotometry, and GraphPad Prism6 software to define a total of 12 aggregation indices (including number of aggregates, aggregated mass index, optical density, aggregation coefficient, span, number of aggregates at plateau time-point, aggregated area index, aggregated diameter index, aggregated symmetry index, time constant, half-life, and rate constant). The data showed linear correlation between crystal concentration and almost all of these indices, except only for rate constant. Among these, number of aggregates provided the greatest regression coefficient (*r* = 0.997; *p* < 0.001), whereas the equally second rank included aggregated mass index and optical density (*r* = 0.993; *p* < 0.001 and *r* = −0.993; *p* < 0.001, respectively) and the equally forth were aggregation coefficient and span (*r* = 0.991; *p* < 0.001 for both). These five indices are thus recommended as the most appropriate indices for quantitative analysis of COM crystal aggregation *in vitro*.

## Introduction

Crystal aggregation has been recognized as an initial important mechanism for kidney stone formation (Christmas et al., 2002). Microcrystals of calcium and other salts can be found in concentrated normal urine or renal tubular fluid. Nevertheless, the individual urinary microcrystals are too small and are normally eliminated by urinary or tubular fluid flow (Kok and Papapoulos, 1993; Christmas et al., 2002; Robertson, 2004). In addition to crystal growth and adhesion of crystals to renal tubular epithelial cells, crystal aggregation is a mechanism that causes the crystals to retain inside renal tubules by their rapidly enlarged size as a result of combining several individual crystals into an agglomerate (Kok and Papapoulos, 1993; Robertson, 2004). Moreover, previous studies have found that deficiencies of stone inhibitors are associated with large aggregated crystals in the urine of stone formers (Robertson et al., 1969; Robertson and Peacock, 1972). In addition, urinary macromolecules (e.g., Tamm-Horsfall protein) (Viswanathan et al., 2011) and various types of bacteria (Chutipongtanate et al., 2013) have been demonstrated to promote crystal aggregation.

Although crystal aggregation is a very important mechanism inducing kidney stone disease, it had been under-investigated in the past because of a limitation of available assays to examine crystal aggregation. Most of the previous studies employed microscopic examination to determine agglomerate of individual crystals to represent crystal aggregation (Thongboonkerd et al., 2008; Chutipongtanate et al., 2013). However, quantitative analysis of crystal aggregation depends only on number of the agglomerates and their size (Thongboonkerd et al., 2008; Chutipongtanate et al., 2013). Spectrophotometry is another method available for studying calcium oxalate monohydrate (COM) crystal aggregation by measuring absorbance or optical density (OD) at λ620 nm after mixing supersaturated solutions of calcium chloride and sodium oxalate (Hess et al., 1995; Baumann et al., 1997; Christmas et al., 2002). The decline of OD at λ620 nm has been used as an indirect parameter to determine degree of crystal aggregation (Hess et al., 2000; Baumann et al., 2011). However, the accuracy of such assay is doubtful as the reduction of the OD is an indirect measurement rather than the direct evidence of crystal aggregation. These limitations reflect the lack of systematic analysis of quantitative assays and indices that can be used to quantify the degree of crystal aggregation.

Accordingly, we aimed to define appropriate aggregation indices to quantify the degree of aggregation of COM crystals, which are the most common chemical composition found in kidney stones (Schubert, 2006). Various concentrations (25–800 μg/ml) of individual COM crystals were resuspended in saturated aggregation buffer and then incubated for 1 h. Subsequently, the aggregated crystals were analyzed by microscopic examination, UV-visible spectrophotometry, and GraphPad Prism6 software to define a total of 12 aggregation indices (including number of aggregates, aggregated mass index, optical density, aggregation coefficient, span, number of aggregates at plateau time-point, aggregated area index, aggregated diameter index, aggregated symmetry index, time constant, half-life, and rate constant). These indices were then systematically compared in correlation with dosage or concentration of the crystals seeded in the saturated aggregation buffer.

## Materials and methods

### COM crystal preparation

Individual COM crystals were freshly prepared as previously described (Thongboonkerd et al., 2006, 2008), by mixing 10.0 mM CaCl_2_·2H_2_O and 1.0 mM Na_2_C_2_O_4_ (1:1 v/v) in a buffer containing 10 mM Tris-HCl and 90 mM NaCl (pH 7.4). The solution was incubated at room temperature (set as 25°C) overnight. COM crystals were then harvested by a centrifugation at 2,000 g for 5 min. The supernatant was discarded, whereas COM crystals were washed 3 times with methanol. After another centrifugation at 2,000 g for 5 min, methanol was discarded and the crystals were air-dried overnight at room temperature.

### Preparation of saturated aggregation buffer

The saturated aggregation buffer was prepared by modifying the plain artificial urine, which was made by dissolving 2.427 g urea, 0.034 g uric acid, 0.090 g creatinine, 0.297 g Na_3_C_6_H_5_O_7_·2H_2_O, 0.634 g NaCl, 0.450 g KCl, 0.161 g NH_4_Cl, 0.089 g CaCl_2_·2H_2_O, 0.100 g MgSO_4_·7H_2_O, 0.034 g NaHCO_3_, 0.003 g NaC_2_O_4_, 0.258 g Na_2_SO_4_, 0.100 g NaH_2_PO_4_·H_2_O, and 0.011 g Na_2_HPO_4_ in 200 ml deionized (18.2 MΩ·cm) water. Then, the artificial urine was made to be “saturated” with calcium and oxalate ions (namely “saturated aggregation buffer”) by adding sufficient amount of COM crystals into the plain artificial urine until the crystals could not be dissolved anymore. The suspension was then filtrated through 0.2-μm cellulose acetate membrane (Sartorius Stedim Biotech; Göttingen, Germany) and the saturated artificial urine was collected and used for all subsequent experiments.

### Crystal aggregation assay

Crystal aggregation assay was performed by seeding individual COM crystals into a well of 6-well plate (Corning Inc.; Corning, NY) containing the saturated aggregation buffer at different concentrations (25, 50, 100, 200, 400, and 800 μg/ml). The suspension was then trembled in a shaking incubator (Zhicheng; Shanghai, China) at 150 rpm, 25°C. After 1-h incubation, crystal morphology was examined and imaged using Nikon Eclipse Ti-S inverted light microscope (Nikon; Tokyo, Japan). Thereafter, the suspension of the aggregated crystals was transferred into a cuvette and subjected to measurement of absorbance (optical density; OD) at λ620 nm using a UV-visible spectrophotometer (Analytik Jena AG; Jena, Germany) with 10-s interval over 300 s.

### Aggregation index calculation

To quantitatively analyze degree of COM crystal aggregation, a total of 12 aggregation indices were calculated from morphological examination, UV-visible spectrophotometry, and analysis with GraphPad Prism6 software (GraphPad Software. Inc.; La Jolla, CA).

From morphological examination, aggregated crystals were defined as an assembly of three or more individual crystals tightly joined together. NIS Element D software version 4.11 (Nikon) was applied to measure number of aggregated crystals, minimal and maximal diameters of aggregated crystals, and crystal area from at least 15 fields per well.

From UV-visible spectrophotometry, the OD values at λ620 nm measured from at least 3 independent experiments were plotted along the *y*-axis against time, which was on the *x*-axis. Plateau time-point was defined as the time-point at which: (i) the ration of OD values at the closest interval time-points was <1±0.05; and (ii) *t*-test of the OD values at the closest interval time-points showed no statistically significant difference (*p* ≥ 0.05).

Finally, the OD values obtained from UV-visible spectrophotometry were entered into GraphPad Prism6 software to calculate for rate constant, half-life, span, and time-constant indices.

From these, indices #1-#8 were manually calculated using the following formulas, whereas indices #9-#12 (rate constant, half-life, span, and time-constant indices) were automatically generated by the GraphPad Prism6 software.

Index #1:

$$\begin{array}{l} \text{Number of aggregates }=\text{ Average number of aggregates per}\\ \text{low}-\text{power field }\left(\text{LPF}\right) \end{array}$$

Index #2:

$$\begin{array}{l} \text{Aggregated diameter index }=\frac{\text{Average maximum widt}{\text{h}}_{\text{aggregate}}}{\text{Average maximum widt}{\text{h}}_{\text{single}}} \end{array}$$

Index #3:

$$\begin{array}{l} \text{Aggregated area index }=\frac{\text{Average are}{\text{a}}_{\text{aggregate}}}{\text{Average are}{\text{a}}_{\text{single}}} \end{array}$$

Index #4:

$$\begin{array}{l} \text{Aggregated mass index }=\text{ Number of aggregates}\\ \;\times \text{ Aggregated area index} \end{array}$$

Index #5:

$$\begin{array}{l} \text{Aggregated symmetry index}=\frac{\text{Maximum widt}{\text{h}}_{\text{aggregate}}}{\text{Minimum widt}{\text{h}}_{\text{aggregate}}} \end{array}$$

Index #6:

$$\begin{array}{l} \text{Optical density }=\text{ OD measured at λ}620\text{ nm} \end{array}$$

Index #7:

$$\begin{array}{l} \text{Aggregated coefficient}=\frac{\text{O}{\text{D}}_{300\text{ s}}-\text{O}{\text{D}}_{0\text{ s}}}{\text{Tim}{\text{e}}_{300\text{ s}}-\text{Tim}{\text{e}}_{0\text{ s}}} \end{array}$$

Index #8:

$$\begin{array}{l} \text{Number of aggregates at plateau time}-\text{point}\\ \;=\text{ Area under curve at plateau time}-\text{point} \end{array}$$

***Note 1:*** “*aggregate” stands for the crystal aggregate, whereas “single” stands for the non-aggregated crystal*.

***Note 2:*** *Indices #9-#12 were automatically generated from the GraphPad Prism6 software*.

### Statistical analysis

All the quantitative data are reported as mean ± SEM of those derived from at least 3 independent experiments. The linear correlation between aggregation index and concentration of the seeded individual COM crystals was tested by Pearson's correlation (SPSS Statistics, version 18.0). This analysis was used to determine the linear regression coefficient (*r*) for validation of the recommended aggregation index. *P* < 0.05 was considered as statistically significance.

## Results and discussion

We aimed to define appropriate aggregation indices to quantify the degree of COM crystal aggregation. Briefly, the aggregated crystals were produced by trembling individual crystals, which were seeded in the saturated aggregation buffer, at 150 rpm for 1 h at room temperature (set at 25°C) to allow crystal-crystal interactions that ultimately led to the formation of aggregation complex. Thereafter, the crystal aggregates were analyzed by microscopic examination, UV-visible spectrophotometry, and GraphPad Prism6 software.

**Figure 1:**
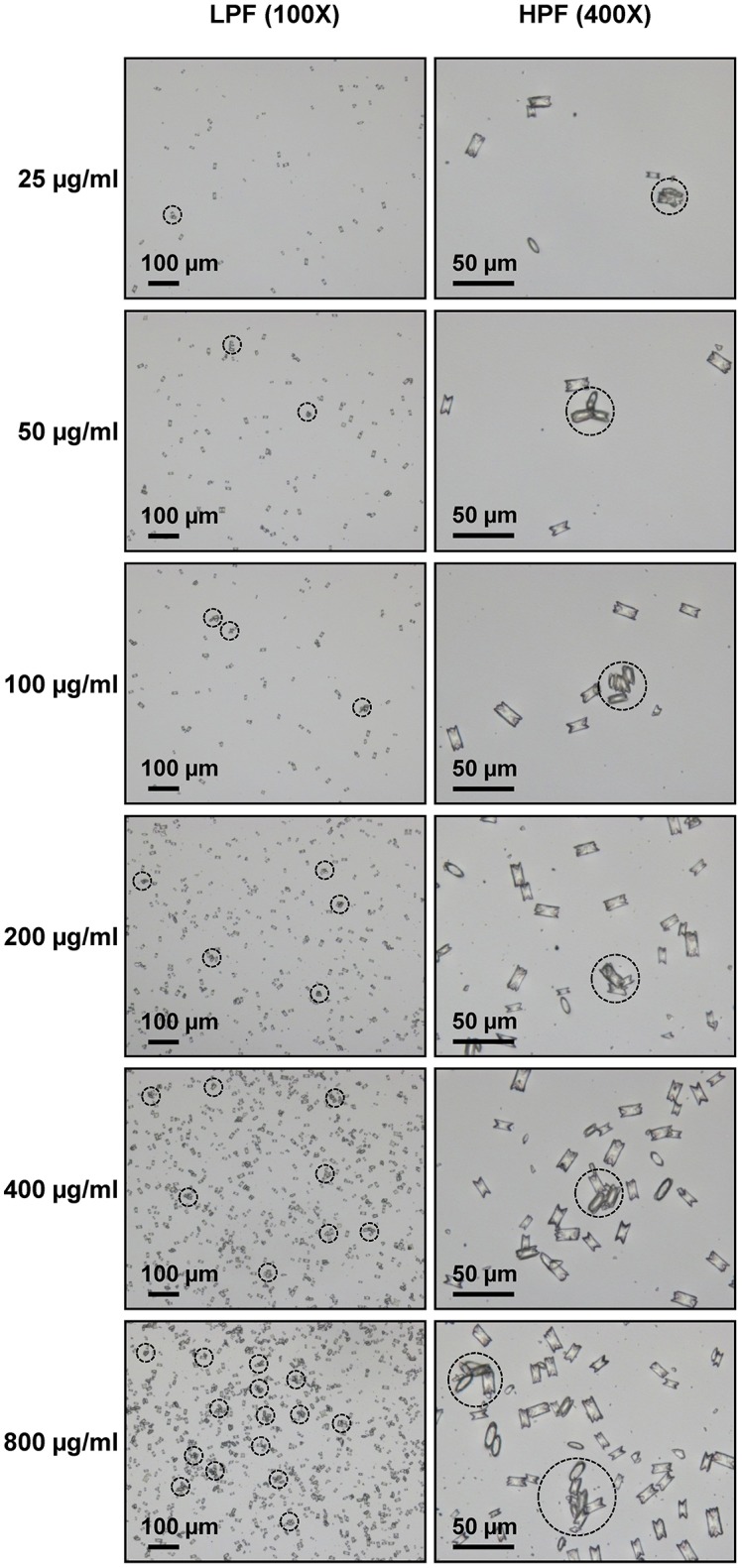
Morphology of COM crystal aggregates. Aggregated COM crystals were derived from various concentrations of the seeded individual COM crystals. Images were taken under Nikon Eclipse Ti-S inverted light microscope (Nikon; Tokyo, Japan) at low-power field (LPF) (100X) and high-power field (HPF) (400X). The dashed circle indicates the crystal aggregate, which was defined as an assembly of three or more individual crystals tightly joined together.

**Figure 2:**
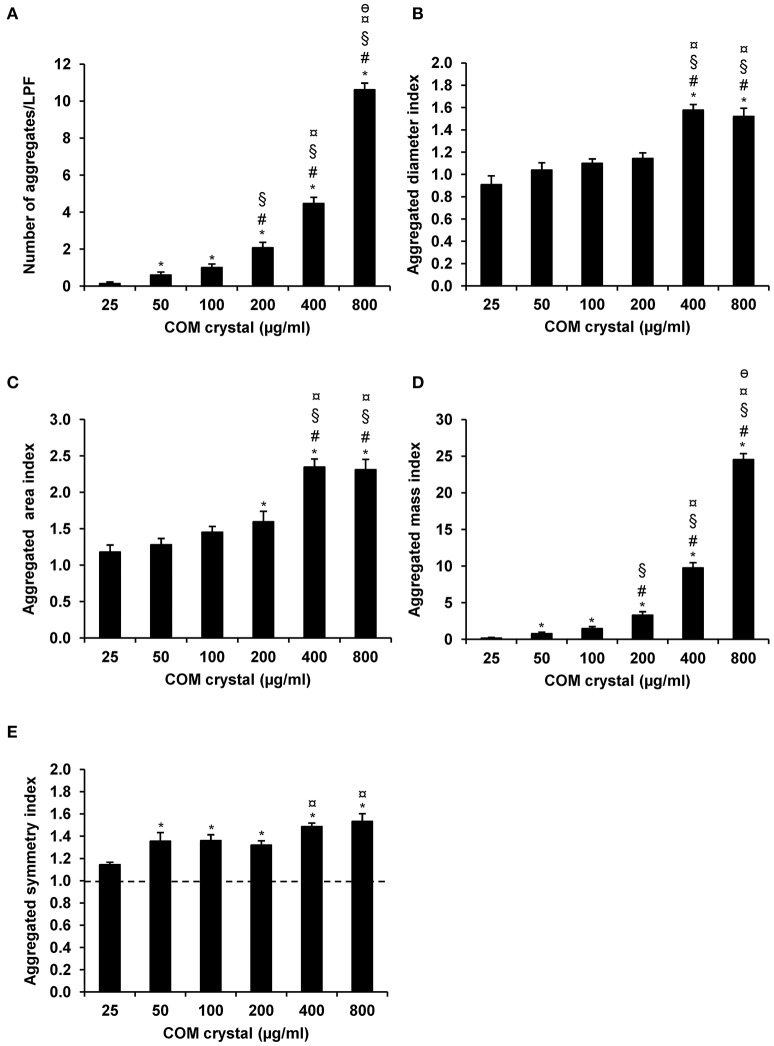
COM crystal aggregation indices derived from microscopic examination. Quantitative analysis following microscopic examination revealed five aggregation indices, including number of aggregates **(A)**, aggregated diameter index **(B)**, aggregated area index **(C)**, aggregated mass index **(D)**, and aggregated symmetry index **(E)**. Each bar represents mean ± SEM of the data obtained from randomized 15 LPF/replicate and 3 independent experiments. ^*^p < 0.05 vs. 25 μg/ml; ^#^*p* < 0.05 vs. 50 μg/ml; ^§^*p* < 0.05 vs. 100 μg/ml; ^¤^*p* < 0.05 vs. 200 μg/ml; and ^θ^*p* < 0.05 vs. 400 μg/ml.

Microscopic examination showed the increasing number and size of the crystal aggregates when the concentration of the seeded individual crystals was increased (Figure 1). These images were then analyzed by NIS Element D software and 5 aggregation indices were obtained from such measurements. Number of aggregates, aggregated diameter index, aggregated area index, aggregated mass index, and aggregated symmetry index were calculated using the formulas detailed in “Materials and Methods.” The data showed increasing of these indices when the concentration of the seeded individual crystals was increased (Figure 2).

**Figure 3:**
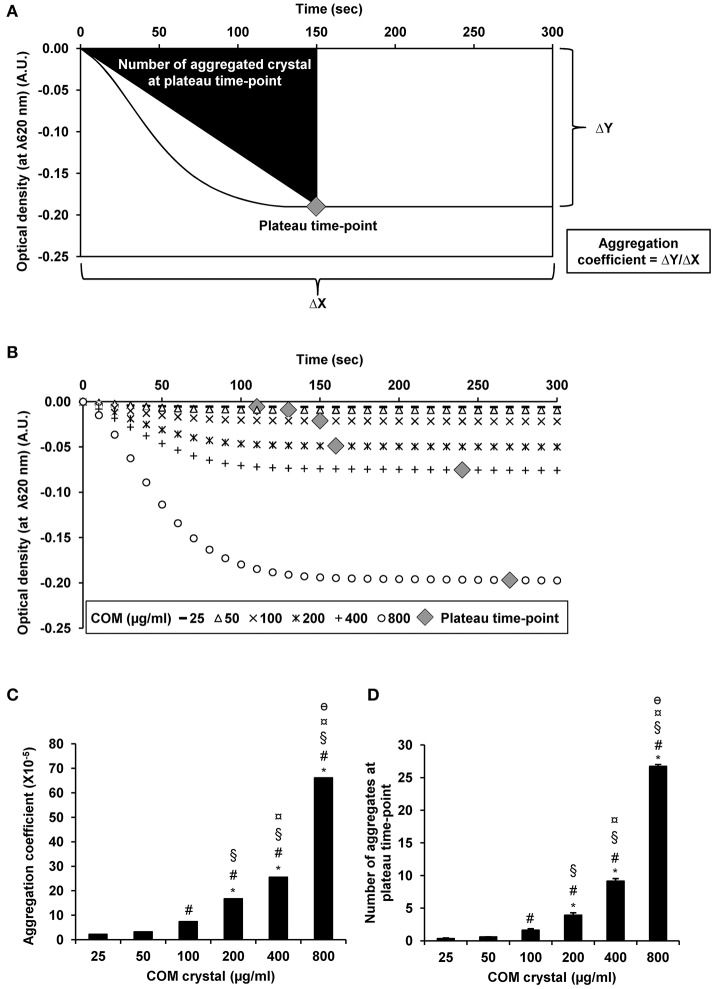
COM crystal aggregation indices derived from UV-visible spectrophotometry. Rationales of defining aggregation indices from UV-visible spectrophotometry are illustrated in **(A)**. Quantitative analysis following UV-visible spectrophotometry revealed three additional aggregation indices, including optical density **(B)**, aggregation coefficient **(C)**, and number of aggregates at plateau time-point **(D)**. Each bar represents mean ± SEM of the data obtained from 3 independent experiments. A.U., arbitrary unit. ^*^p < 0.05 vs. 25 μg/ml; ^#^*p* < 0.05 vs. 50 μg/ml; ^§^*p* < 0.05 vs. 100 μg/ml; ^¤^*p* < 0.05 vs. 200 μg/ml; ^θ^*p* < 0.05 vs. 400 μg/ml.

From UV-visible spectrophotometric analysis, the absorbance (optical density; OD) of the aggregated crystal suspension was serially measured at λ620 nm every 10 s for a total of 300 s. The rationales of obtaining aggregation coefficient and defining the plateau time-point are illustrated in Figure 3A. The data showed that the optical density was inversely correlated with the concentration of the seeded individual crystals (Figure 3B). Our data were consistent with those reported in previous studies, which demonstrated that the higher degree of aggregation was correlated with the greater decline of the optical density in an inverse manner (Baumann et al., 2010). However, aggregation coefficient and number of the aggregates at plateau time-point were directly correlated with the concentration of the seeded individual crystals (Figures 3C,D, respectively).

**Figure 4:**
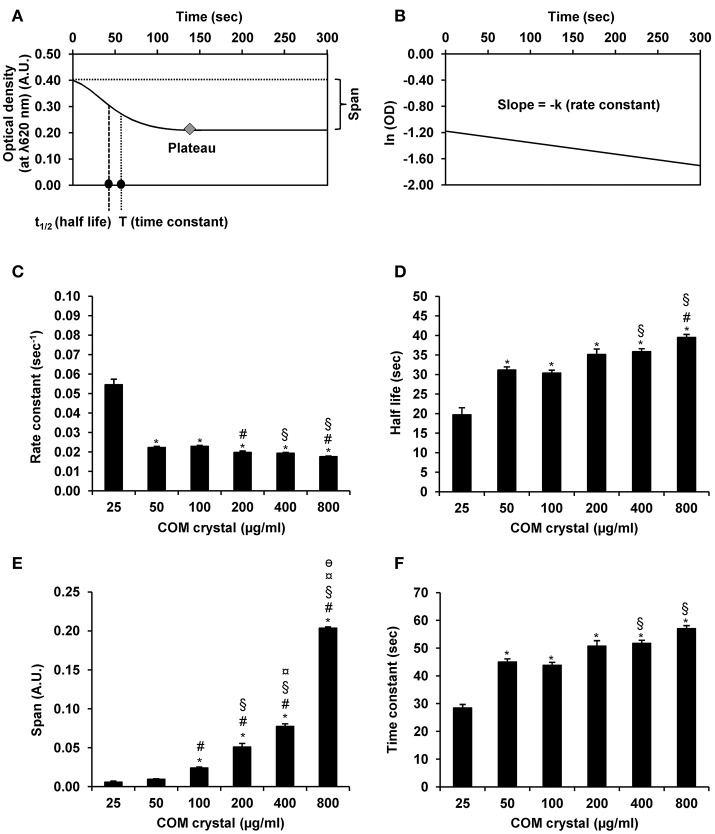
COM crystal aggregation indices derived from GraphPad Prism6 software. Rationales of defining aggregation indices from GraphPad Prism6 software are illustrated in **(A,B)**. Quantitative analysis using GraphPad Prism6 software revealed four additional aggregation indices, including rate constant **(C)**, half-life **(D)**, span **(E)**, and time constant **(F)**. Each bar represents mean ± SEM of the data obtained from 3 independent experiments. A.U., arbitrary unit. ^*^p < 0.05 vs. 25 μg/ml; ^#^*p* < 0.05 vs. 50 μg/ml; ^§^*p* < 0.05 vs. 100 μg/ml; ^¤^*p* < 0.05 vs. 200 μg/ml; ^θ^*p* < 0.05 vs. 400 μg/ml.

Furthermore, OD values derived from all measurements by UV-visible spectrophotometry were also submitted to GraphPad Prism6 software for defining other aggregation indices, including rate constant, half-life, span, and time constant. The rationales of obtaining these indices are summarized in Figures 4A,B. All these additional aggregation indices demonstrated the tendency of dose-dependent increase of these indices along with the increasing dosage of the seeded individual crystals (Figures 4D–F), except only for the rate constant that did not show such trend (Figure 4C).

**Figure 5:**
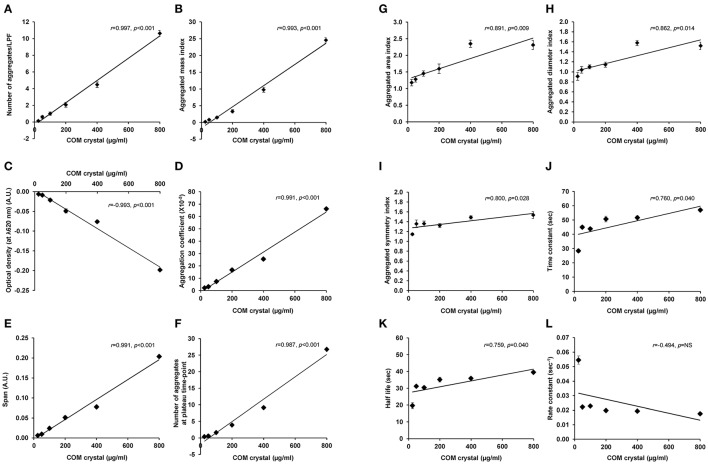
Pearson's correlation analysis of each aggregation index and concentration of the seeded individual COM crystals. Pearson's correlation analysis (SPSS Statistics, version 18.0) was performed to evaluate the linear correlation between concentration of the seeded individual COM crystals and number of aggregates **(A)**, aggregated mass index **(B)**, optical density **(C)**, aggregation coefficient **(D)**, span **(E)**, number of aggregates at plateau-time point **(F)**, aggregated area index **(G)**, aggregated diameter index **(H)**, aggregated symmetry index **(I)**, time constant **(J)**, half-life **(K)**, and rate constant **(L)**. Each data point represents mean ± SEM of the data obtained from 3 independent experiments. A.U., arbitrary unit; NS, not statistically significant.

Finally, Pearson's correlation analysis was performed to validate the tendency of correlation observed from all measurements as detailed above. The data showed linear correlation of almost all of these indices (except only for rate constant) with crystal concentration (Figure 5 and Table 1). Among these, number of aggregates provided the greatest regression coefficient (*r* = 0.997; *p* < 0.001), whereas the equally second rank included aggregated mass index and optical density (*r* = 0.993; *p* < 0.001 and *r* = −0.993; *p* < 0.001, respectively) and the equally forth were aggregation coefficient and span (*r* = 0.991; *p* < 0.001 for both) (Figure 5 and Table 1).

**Table 1:** Pearson's analysis of correlation between concentration of the seeded individual COM crystals and each aggregation index.

**Rank**	**Aggregation index**	**Pearson's analysis**	
		***r***	***P*-value**
1^***^	Number of aggregates	0.997^***^	<0.001
2^**^	Aggregated mass index	0.993^**^	<0.001
2^**^	Optical density	−0.993^**^	<0.001
4^*^	Aggregation coefficient	0.991^*^	<0.001
4^*^	Span	0.991^*^	<0.001
6	Number of aggregates at plateau time-point	0.987	<0.001
7	Aggregated area index	0.891	0.009
8	Aggregated diameter index	0.862	0.014
9	Aggregated symmetry index	0.800	0.028
10	Time constant	0.760	0.040
11	Half-life	0.759	0.040
12	Rate constant	−0.494	NS

*Ranked by the linear regression coefficient (r)*.

*NS, Not statistically significant*.

*** *The first rank with the greatest linear regression coefficient (r)*.

** *The same second rank with identical r*.

* *The same forth rank with identical r*.

Herein, we have developed a simple method for quantitative analysis of COM crystal aggregation. The controllable and measurable seeded COM crystals were used as the starting materials for determining and measuring crystal aggregation. In contrast, the methods described in other previous studies mostly generated crystal aggregation directly from mixing CaCl_2_ with Na_2_C_2_O_4_ in a cuvette without determination of the saturation of such suspension prior to crystal aggregation assay (Kulaksizoglu et al., 2007). Having done as described in the previous studies, the degree and amount of crystal aggregates were hard to be controlled and measurement of crystal aggregation, thus, could be erroneous (Kulaksizoglu et al., 2007). Moreover, the crystal aggregation could be mixed up with (neo-) crystallization and nucleation. Using our method, in which the aggregation buffer was made to be saturated with calcium and oxalate ions before the seeded individual COM crystals were added, neocrystallization could be excluded and measurements of the amount and degree of crystal aggregates should be more precise. Also, our strategy of using this saturated aggregation buffer together with the fixed and controllable amount of the seeded COM crystals was that we did not want to have any effects from dissolution (such as when the crystals were seeded into other solutions, for example plain artificial urine or even deionized water, without saturation of calcium and oxalate ions) that can affect crystal sizes because sizes of COM crystals can affect crystal aggregation (Gan et al., 2016).

In addition, we intended to simulate the physiological condition of kidney stone formation in the urinary tract. Many lines of evidence have shown that patients with calcium oxalate kidney stones (stone formers) frequently have supersaturation of calcium and oxalate ions in their urine (Parks et al., 1997; Lingeman et al., 1999; Coe et al., 2001). Thus we attempted to mimic the *in vivo* model for studying kidney stone formation mechanisms. Moreover, various protocols of the saturated buffers have been used and reported for evaluation of COM crystal aggregation (Hess et al., 1989; Wesson et al., 2005; Viswanathan et al., 2011), similar to our saturated aggregation buffer to reduce the crystal dissolution.

Our method is also easier to perform, as it does not need to continuously stir the suspension inside a small cuvette by using magnetic mini-bar as in the case of the previous protocols (Kulaksizoglu et al., 2007). Continuous stirring may be associated with alterations in size of individual crystals and their aggregates. This can generate variations during analysis of the crystal aggregates, and thus making quantitative analysis of crystal aggregation easily erroneous in the past. Furthermore, we performed this assay by gentle trembling of the seeded crystals in the saturated aggregation buffer that can mimic the turbulent flow inside renal tubules and calyceal system of the kidney, whereas the protocols established previously might apply a more rigorous force that were not the normal physiologic condition to generate crystal aggregates.

Nevertheless, limitations of our approach should be also noted. Our measurements were done entirely “*in vitro*” that might not represent all the “*in vivo*” events of COM crystal aggregation and kidney stone formation. In the kidney, the co-occurrence of crystal aggregation and neocrystallization can be expected. In the present study, we attempted to precisely define the most appropriate index to measure degree of COM crystal aggregation without any effects from neocrystallization that might not be applicable to the *in vivo* phenomena. Moreover, although COM is the most common type of kidney stones, a mixture of crystal types in one stone former (patient) can be also expected. These confounding factors should be concerned when these assays and indices are to be applied for *in vivo* study.

In conclusion, we have defined simple and reproducible aggregated indices for *in vitro* quantitative analysis of COM crystal aggregation. Among all 12 indices calculated, number of aggregates, aggregated mass index, optical density, aggregation coefficient, and span are highly recommended for practical use in quantitative analysis of the crystal aggregation in kidney stone research.

## Author contributions

SC and VT designed research; SC performed experiments; SC and VT analyzed data; SC and VT wrote the manuscript; All authors reviewed the manuscript.

### Conflict of interest statement

The authors declare that the research was conducted in the absence of any commercial or financial relationships that could be construed as a potential conflict of interest.
